# The Role of Interleukin-23 in the Early Development of Emphysema in HIV1^+^ Smokers

**DOI:** 10.1155/2016/3463104

**Published:** 2016-06-29

**Authors:** Igor Z. Barjaktarevic, Ronald G. Crystal, Robert J. Kaner

**Affiliations:** ^1^Division of Pulmonary and Critical Care Medicine, Weill Medical College of Cornell University, New York, NY 10065, USA; ^2^Department of Genetic Medicine, Weill Medical College of Cornell University, New York, NY 10065, USA

## Abstract

*Rationale.* Matrix metalloproteinase-9 (MMP-9) expression is upregulated in alveolar macrophages (AM) of HIV1^+^ smokers who develop emphysema. Knowing that lung epithelial lining fluid (ELF) of HIV1^+^ smokers contains increased levels of inflammatory cytokines compared to HIV1^−^ smokers, we hypothesized that upregulation of lung cytokines in HIV1^+^ smokers may be functionally related to increased MMP-9 expression.* Methods.* Cytokine arrays evaluated cytokine protein levels in ELF obtained from 5 groups of individuals: HIV1^−^ healthy nonsmokers, HIV1^−^ healthy smokers, HIV1^−^ smokers with low diffusing capacity (DL_CO_), HIV1^+^ nonsmokers, and HIV1^+^ smokers with low DL_CO_.* Results*. Increased levels of the Th17 related cytokine IL-23 were found in HIV1^−^ smokers with low DL_CO_ and HIV1^+^ smokers and nonsmokers. Relative IL-23 gene expression was increased in AM of HIV1^+^ individuals, with greater expression in AM of HIV1^+^ smokers with low DL_CO_. Infection with HIV1* in vitro* induced IL-23 expression in normal AM. IL-23 stimulation of AM/lymphocyte cocultures* in vitro* induced upregulation of MMP-9. Lung T lymphocytes express receptor IL-23R and interact with AM in order to upregulate MMP-9.* Conclusion*. This mechanism may contribute to the increased tissue destruction in the lungs of HIV1^+^ smokers and suggests that Th17 related inflammation may play a role.

## 1. Introduction 

Survival of individuals infected with HIV1 has been dramatically improved since the introduction of highly active antiretroviral therapy (HAART), but this increased survival has been associated with the development of chronic disorders [1]. One example is the early development of emphysema in HIV1^+^ smokers, with a greater prevalence compared to smokers who are HIV1^−^ [2–5]. Relevant to the pathogenesis of this HIV1^+^ smoker-associated early emphysema, alveolar macrophages (AM) of HIV1^+^ smokers release increased levels of matrix metalloproteinases (MMPs), enzymes thought to play a key role in the pathogenesis of emphysema by virtue of their ability to degrade extracellular matrix and basement membrane components and modulate the recruitment of leukocytes into the lung [6–8].

In the context that lung epithelial lining fluid (ELF) of HIV1^+^ smokers has increased levels of a variety of cytokines [5, 7–10] and that several cytokines mediate the activation state of AM [11, 12], we hypothesized that, in the presence of cigarette smoke, upregulation of MMP-9 is associated with increased levels of proinflammatory cytokines and that their interplay may be contributing to the early development of emphysema in HIV1^+^ smokers. Based on recent studies demonstrating the importance of IL-23 and the Th17 immune response in the development of chronic obstructive pulmonary disease [13–15], their role in the progression of HIV-1 infection [16–20], and the effect of IL-23 on increased lung MMP-9 in mice [21], we focused on a possible role of IL-23 in the upregulation of MMP-9 in AM of HIV1^+^ smokers. Assessment of inflammatory/immune cells recovered by lavage and lower respiratory tract ELF of HIV1^−^ healthy nonsmokers, HIV1^+^ nonsmokers, HIV1^−^ healthy smokers, HIV1^−^ smokers with low DL_CO_ and HIV1^+^ smokers with low DL_CO_ demonstrates that the upregulation of the MMP-9 by AM in HIV^+^ smokers is likely derived, at least in part, by AM expression of IL-23 working in concert with T lymphocytes to induce AM to express MMP-9, a known contributor to the pathogenesis of emphysema [22].

## 2. Methods

### 2.1. Human Subjects

All individuals were evaluated at the Weill Cornell NIH Clinical and Translational Science Center and the Department of Genetic Medicine Clinical Research Facility, using Institutional Review Board-approved clinical protocols. Individuals underwent an initial screening evaluation including history, complete physical exam, blood studies, urine analysis, chest X-ray, pulmonary function tests, and electrocardiogram. Individuals with any significant prior use of addictive drugs (other than nicotine) in the previous 6 months were excluded. Blood studies included a complete blood count, coagulation parameters, serum electrolytes, liver and kidney function tests, serum evaluation for human immunodeficiency virus antibodies, HIV1 viral load, CD4 count, hepatitis profile (A, B, and C), anti-nuclear antibodies, sedimentation rate, and rheumatoid factor. Current smoking status was verified by measurement of urinary levels of nicotine and its derivative cotinine and blood carboxyhemoglobin. Pulmonary function tests were carried out according to American Thoracic Society guidelines [21, 23–26]. Individuals who smoked and had a diffusing capacity < 80% predicted were further evaluated with noncontrast high resolution computed tomography (CT) of the chest. The 5 subject groups were defined as follows (Table 1). For all groups, no subjects had active pulmonary symptoms or a history of lung infections.

#### 2.1.1. HIV1^+^ Smokers with Low DL_CO_

This group (*n* = 11) included individuals who were HIV1^**+**^ and were current smokers. All subjects reported taking HAART. Each of these individuals had lung function studies with normal forced expiratory volume in 1 sec (FEV1), forced vital capacity (FVC), FEV1/FVC, and total lung capacity (TLC), but with diffusing capacity < 80% predicted as a physiological correlate of early emphysema [27, 28]. Assessment of the chest high-resolution CT (HRCT) of this group had visual evidence of minimal to mild emphysema in 8/11 subjects confirmed by two experienced pulmonary physicians and a radiologist who was blinded to the subjects' history.

#### 2.1.2. HIV1^−^ Smokers with Low DL_CO_

This group (*n* = 18) were characterized by the same pulmonary function as for the HIV1^**+**^ smokers. A chest HRCT was available for the majority of subjects in the study group and revealed visual changes of minimal to mild emphysema in 9/14.

#### 2.1.3. HIV1^−^ Healthy Smokers

These individuals (*n* = 32) included current smokers with a normal screening evaluation, normal pulmonary function tests and chest X-ray, and a positive urine screen for smoking.

#### 2.1.4. HIV1^−^ Healthy Nonsmokers

These individuals (*n* = 17) were lifelong nonsmokers with a normal screening evaluation, normal pulmonary function tests and chest X-ray, and a negative urine screen for smoking.

#### 2.1.5. HIV1^+^ Nonsmokers

This group (*n* = 5) consisted of lifelong HIV1^**+**^ nonsmokers with an otherwise normal screening evaluation, normal pulmonary function tests and chest X-ray, and a negative urine screen for smoking.

For the* in vitro* analyses, each assay was performed on a random subset of the overall study group; the number of subjects used for each analysis is indicated.

### 2.2. Collection of Alveolar Macrophage/Lymphocyte Cocultures

Fiberoptic bronchoscopy was performed to obtain inflammatory/immune cells and ELF from the lower respiratory tract using bronchoalveolar lavage, as previously described [8]. The lavage fluid was filtered through 2 layers of gauze and centrifuged at 1250 rpm for 5 min, 4°C. The supernatant was aliquoted and stored at −80°C. Cells were suspended in 5 mL Ack Lysing Buffer (Invitrogen*™*, Grand Island, NY) and then washed twice in RPMI 1640 containing 10% fetal bovine serum, 50 U/mL penicillin, 50 *μ*g/mL streptomycin, and 2 mM glutamine (Invitrogen). Cell viability was estimated by trypan blue exclusion and expressed as a percentage of the total cells recovered. Total cell number was determined by counting in a hemocytometer. Cells were suspended in media (10^6^ cells/mL) and an aliquot of 250 *μ*L with 250 *μ*L fetal bovine serum (Invitrogen) was used for a differential cell count assessed by cytocentrifugation (Cytospin 11; Shandon Instruments, Pittsburgh, PA) and stained with DiffQuik (Baxter Healthcare, Miami, FL). Cells recovered from BAL fluid were seeded in 12-well plastic culture dishes (10^6^/well) and alveolar macrophages (AM) were enriched by adherence for 12 hr at 37°C in a 5% CO_2_ humidified incubator. Nonadherent cells were removed by thorough washing with RPMI 1640. AM represented ≥95% of cells, consistent with published literature [29]. The remaining cells consisted of lymphocytes, neutrophils, NK cells, or eosinophils as assessed by flow cytometric analysis (not shown). Because this adherence method for AM isolation is known to contain a small number of lymphocytes, we refer to the cultures as AM/lymphocyte cocultures.

### 2.3. Cell Cultures

All cells including AM, T cells recovered from the lower respiratory tract, and T cells from blood were cultured from 6 h to 48 h at 37°C in a 5% CO_2_ humidified incubator in RPMI 1640 medium supplemented with 10% fetal bovine serum, 50 U/mL penicillin, 50 *μ*g/mL streptomycin, and 2 mM glutamine (Invitrogen). Cell viability was estimated by trypan blue exclusion and expressed as % total cells.

### 2.4. Cytokine Protein Arrays

Lavage fluid supernatant was concentrated 10-fold on Centricon filters (Millipore, Billerica, MA). The concentrated supernatant was assessed for 36 cytokines with a Human Cytokine Array Panel A (R&D Systems, Minneapolis, MN). The supernatant was mixed with a cocktail of biotinylated detection antibodies and incubated on a nitrocellulose membrane, embedded with cognate capture antibodies. Arrays were then treated with streptavidin-horseradish peroxidase for 30 min. Chemiluminescent detection reagents were added (GE Healthcare, Piscataway, NJ) and the array was developed. The signal present was proportional to the amount of cytokine captured. Optical densities of obtained values were measured and quantified using Image J software.

### 2.5. TaqMan PCR

Relative gene expressions of IL-23, IL-23R, and MMP-9 were evaluated by measuring specific mRNA levels via TaqMan real-time reverse transcriptase (RT) PCR analysis. Total RNA was extracted using a modified version of the TRIzol method (Invitrogen, Grand Island, NY), in which RNA is purified directly from the aqueous phase (RNeasy MinElute RNA purification kit, Qiagen, Valencia, CA). RNA samples were stored in RNA Secure (Ambion, Austin, TX) at −80°C. RNA integrity was determined by running an aliquot of each RNA sample on an Agilent Bioanalyzer (Agilent Technologies, Palo Alto, CA). The concentration was determined using a NanoDrop ND-1000 spectrophotometer (NanoDrop Technologies, Wilmington, DE). A first-strand cDNA was synthesized from 2 *μ*g of total RNA in 50 *μ*L reaction volume, using the TaqMan Reverse Transcriptase Reaction Kit (Applied Biosystems, Foster City, CA), with random hexamers as primers. The primers specific for each mRNA (MMP-9: hs00234579_m1, IL-23a: hs00900829_g1, and IL-23R: hs00332759_m1) and the endogenous controls human *β*-2-microglobulin (VIC®/MGB Probe, Primer Limited) and human *β*-actin endogenous control (VIC/MGB Probe, Primer Limited) were purchased from Applied Biosystems. For each individual sample, two conditions were used: 1 : 10 and 1 : 100 dilution of the cDNA reaction, and each dilution was assayed in triplicate wells. The PCR reactions were run in an Applied Biosystems Sequence Detection System 7700. The threshold cycles (Cts) were calculated as an average of the triplicate reactions for each condition, and the ΔCt was calculated for each sample using the rRNA as an endogenous reference. ΔΔCt was calculated by subtracting the calibrator from the ΔCt in each individual sample using the algorithm provided by Applied Biosystems.

### 2.6. Cytokine Treatment of AM/Lymphocyte Cocultures

After removal of nonadherent cells from the lavage fluid, cultured AM containing small numbers of lymphocytes from both HIV1^+^ and HIV1^−^ individuals were incubated with RPMI media in 12-well plates at 37°C in a 5% CO_2_ humidified incubator. AM were treated with serum-free media alone or with serum-free media containing rhIL-23 (R&D systems) at various concentrations. Treated AM/lymphocytes were cultured overnight for quantitative cDNA analysis or 48 hours for the analysis of substrate (MMP-9) concentration in conditioned media.

### 2.7. Gelatin Zymography

For analysis of MMP-9 activity, gelatin zymography was carried out by loading equal amounts of protein per sample onto 8% sodium dodecyl sulfate polyacrylamide gels impregnated with 0.1% gelatin (Invitrogen) and separated using nondenaturing electrophoresis. After electrophoresis, the gels were soaked in renaturing buffer with 2.7% Triton X-100 in distilled water (Invitrogen). Gels were then developed and stained with Colloidal Blue (Invitrogen) which reveals the enzymatic activity with white bands against a dark background. Recombinant MMP-9 protein in latent and active forms (Chemicon, Temecula, CA) was used as a positive control. Optical densities of gel zymograms were quantified with Image J software.

### 2.8. HIV1^−^ Infection of Normal AM

HIV1^−^ AM (2-3 × 10^5^/well) were infected with the HIV-1 laboratory strain, JRFL, at 10^3^ median tissue culture infectious doses (TCID50) per well, a dose known to infect cells of monocytic origin [30]. After 12 days of incubation at 37°C, the cells were washed and the RNA was isolated. Productive HIV1 infection by JRFL HIV1 was determined by quantifying p24 antigen in the media supernatant by ELISA (Beckman Coulter, Miami, FL) at 0–12 days after HIV1 JRFL infection.

### 2.9. T Cell Isolation

T cells used in functional experiments (T cells added to cultures of AM) were isolated directly from BAL using T cell rosetting with neuraminidase-treated sheep red blood cell as per standard protocol [31, 32]. T cells used for cDNA analysis were isolated directly from BAL via positive selection using Dynabeads® CD2 Pan T (Invitrogen Dynal, Life Technologies, Grand Island, NY) as per manufacturer's recommendations. T cells isolated by this method had purity > 80% and were used for cDNA quantitative PCR analysis.

### 2.10. AM Purification Using CD68

Purification of AM was based on positive selection of cells expressing CD68, a glycoprotein expressed intracellularly as well as on the surface of macrophages but not on lymphocytes [33]. DSB-X labeled (Molecular Probes, Inc. Eugene, OR) LEAF mouse anti-human anti-CD68 antibody (BioLegend, San Diego, CA) was used to attach to CD68+ AM. Addition of FlowComp Flexi detachable beads (Invitrogen Dynal, Life Technologies, Grand Island, NY) was used to positively isolate CD68+ AM. Magnetic beads were detached using streptavidin reagent as per the manufacturer's recommendations. Estimated purity of positively selected cells prior to the detachment of magnetic beads was estimated to be >90%.

### 2.11. Flow Cytometry

Cells directly isolated from BAL or cultured AM/lymphocytes isolated by adherence were detached from plates using ethylenediaminetetraacetic acid (EDTA; 5 mM) for 3 min with vigorous washing and were thereafter washed with 0.1% bovine serum albumin (BSA). The cells were then labeled with monoclonal anti-CD-68-PE and anti-human IL-23R-PE (R&D Systems) at 4°C for 30 min. The cells were then fixed and permeabilized and immediately analyzed by flow cytometry. Data on a minimum of 20,000 cell fluorescence events were acquired and analyzed using a FACSCalibur instrument (Becton-Dickinson, San Jose, Calif) and CellQuest*™* software. Immediately prior to the analysis, AM were quenched with Crystal violet 0.5% [34].

### 2.12. Affymetrix Microarrays

Analysis was performed using Affymetrix (Santa Clara, CA) microarray HG-U133 Plus 2.0 and associated protocols. An aliquot of each RNA sample was run on an Agilent Bioanalyzer (Agilent Technologies, Palo Alto, CA) to visualize and quantify the degree of RNA integrity. The concentration was determined using a NanoDrop ND-1000 spectrophotometer (NanoDrop Technologies, Wilmington, DE, USA). Strict quality control criteria were used for an RNA sample to be accepted for further processing [35]. Double-stranded cDNA was synthesized from 3 *μ*g of total RNA using the GeneChip One-Cycle cDNA Synthesis Kit, followed by cleanup with GeneChip Sample Cleanup Module,* in vitro* transcription reaction using the GeneChip IVT Labeling Kit, and cleanup and quantification of the biotin-labeled cRNA yield by spectrophotometric analysis. All kits were from Affymetrix. Hybridizations to test chips and to microarrays were performed according to Affymetrix protocols, and microarrays were processed by the Affymetrix fluidics station and scanned with the Affymetrix GeneChip Scanner 3000 7G. The overall microarray quality was verified by the criteria: (1) 3′/5′ ratio for GAPDH < 3; (2) scaling factor range no more than 2.5 standard deviations (SD) from the mean for all microarrays; and (3) expression level for all 100 housekeeping genes (as defined by Affymetrix, http://www.affymetrix.com/estore/) with coefficient of variation of <40%. After scanning, the data on each individual microarray were scaled to an arbitrary target intensity as recommended by Affymetrix, using the Microarray Suite version 5.0 software.

### 2.13. RNA Sequencing

Analysis of the interleukin-23 receptor (IL-23R) gene was carried out using a database of massive parallel sequencing (RNA-seq) of the transcriptome of AM of HIV1^−^ nonsmokers. The resultant reads were aligned to* Homo sapiens* high coverage assembly GRCh37 using Bowtie v 0.12. Reads per kilobase of exon model per million mapped reads (RPKM) were used to quantify transcript levels of the gene.

### 2.14. Statistics

Results of ELF cytokine protein arrays were expressed as mean values of protein concentration ± standard deviation relative to an array internal standard and were displayed on a logarithmic scale. The significance of differences of mean values of demographic data between the 5 groups was analyzed by ANOVA. TaqMan analysis results were expressed as a relative gene expression in comparison to control and/or the lowest expression in particular experiments. The significance of differences in relative gene expression between different phenotypes was tested via ANOVA or Kruskal-Wallis test when the number of compared groups was >2; Student *t*-test or Wilcoxon Signed-Rank Test was used to compare relative gene expression when 2 groups were compared. *p* < 0.05 was considered significant.

## 3. Results

### 3.1. Cytokine Profile of Epithelial Lining Fluid of HIV1^+^ Smokers with Low Diffusing Capacity

A multicytokine array was used to evaluate ELF levels in 27 individuals, including HIV1^+^ and HIV1^−^ nonsmokers, smokers, and smokers with low DL_CO_ (see Table 1 for demographic data). As expected, a relatively small number of cytokines were detectable in the ELF of HIV1^−^ healthy smokers and HIV1^−^ healthy nonsmokers. In contrast, smokers with low DL_CO_ independent of their HIV1 status had a significantly higher proportion of detectable cytokines (Supplemental Figure 1; see Supplemental Table I for definitions of all cytokines in the arrays, at Supplementary Material available online at http://dx.doi.org/10.1155/2016/3463104).

**Figure 1:**
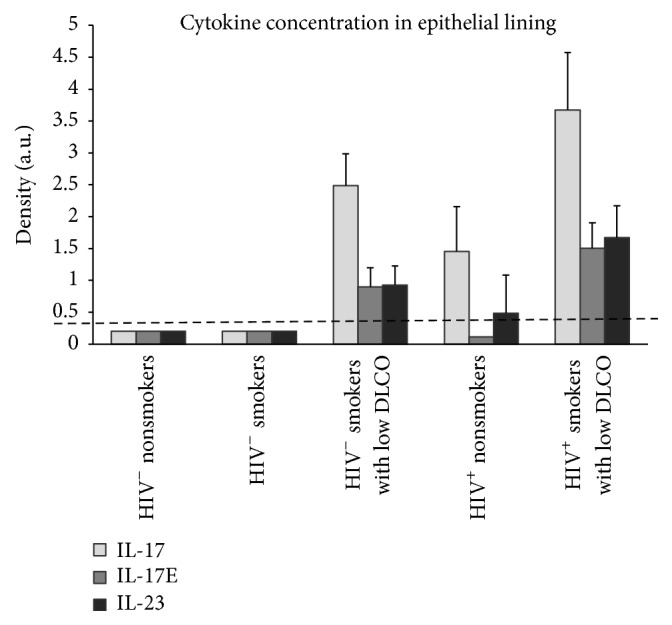
The levels of Th17 cytokines in epithelial lining fluid were analyzed with the Proteome Profiler*™* Human Cytokine Array Panel and average values of HIV1^−^ healthy nonsmokers (*n* = 6), HIV1^−^ healthy smokers (*n* = 7), HIV1^−^ smokers with low DLCO (*n* = 6), HIV1^+^ nonsmokers (*n* = 4), and HIV1^+^ smokers with low DLCO (*n* = 4) are presented in the graph.

In stark contrast to HIV1^−^ nonsmokers or HIV1^−^ healthy smokers, HIV1^+^ nonsmokers had detectible amounts of more than 2/3 of the cytokines represented on the array and more than twice as many compared to HIV1^−^ healthy nonsmokers. Interestingly, the cytokine profiles of HIV1^+^ nonsmokers, HIV1^−^ smokers with low DL_CO_, and HIV1^+^ smokers with low DL_CO_ were very similar and were characterized by the presence of IFN-*γ* as well as the Th17 related cytokines IL-17 and IL-23 that were not present in HIV1^−^ nonsmokers or HIV1^−^ smokers (Figure 1).

These data support the concept that proinflammatory cytokines are increased in the lungs of smokers with low DL_CO_, in both HIV1^**+**^ and HIV1^−^ individuals. HIV1^**+**^ infection itself leads to a broad upregulation of cytokines detectable in ELF, even in the absence of smoking. The presence of IL-23 and IL-17 that characterizes HIV1^+^ individuals independent of their smoking status, as well as HIV1^−^ smokers with low DL_CO_, suggests a potential role of Th17 cellular immune responses in the pathogenesis of emphysema in HIV1^+^ smokers.

In addition to IFN-*γ* and Th17 cytokines, the cytokine profile of HIV1^+^ smokers with low DL_CO_ shared other features with HIV1^+^ nonsmokers and HIV1^−^ smokers with low DL_CO_ including the presence of chemokine (C-C motif) ligand 1 (CCL-1) and chemokine (C-X-C motif) ligand 11 (CXCL-11), both chemotactic for activated T cells [36, 37]. The presence of both of these chemokines is consistent with a role for adaptive immunity and T cells in the early development of emphysema in HIV1^+^ smokers.

### 3.2. IL-23 Expression in Human Alveolar Macrophages

**Figure 2:**
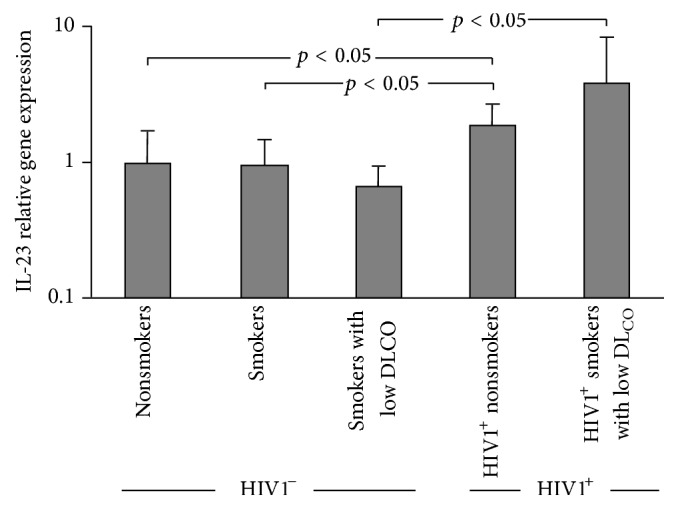
IL-23 expression in AM of HIV1^−^ and HIV1^+^ smokers. Shown is TaqMan PCR analysis of IL-23 expression in AM of HIV1^−^ nonsmokers (*n* = 5), HIV1^−^ smokers (*n* = 5), HIV1^−^ smokers with low DLCO (*n* = 4), HIV1^+^ nonsmokers (*n* = 4), and HIV1^+^ smokers with low DLCO (*n* = 4). Values represent mean relative gene expression in each phenotype compared to mean IL-23 expression in nonsmokers. *p* values shown are based on the Kruskal-Wallis test.

To evaluate whether the IL-23 gene was expressed in human AM, relative expression of AM IL-23 was quantified in a subset of all phenotypes using TaqMan real-time RT-PCR. AM IL-23 expression was detected in all phenotypes. IL-23 was upregulated in both HIV1^+^ phenotypes compared to HIV1^−^ phenotypes (HIV1^−^ nonsmokers (*n* = 5), HIV1^−^ smokers (*n* = 5), HIV1^+^ nonsmokers (*n* = 4), HIV1^−^ smokers with low DL_CO_ (*n* = 4), and HIV1^+^ smokers with low DL_CO_ (*n* = 4; *p* < 0.05, Kruskal-Wallis; Figure 2)). These data are consistent with the results of HG-U133 Plus 2.0 gene microarray analysis where IL-23 expression in AM from HIV1^+^ smokers with low DL_CO_ (*n* = 11) was significantly higher compared to its expression in HIV1^−^ smokers with low DL_CO_ (*n* = 23; not shown). In contrast, there was no significant difference in the extent of upregulation of IL-23 in smoking compared to nonsmoking phenotypes. These data indicate that HIV1 infection correlates with the increased AM expression of IL-23.

### 3.3. Effect of* In Vitro* HIV1 Infection on AM IL-23 Expression

**Figure 3:**
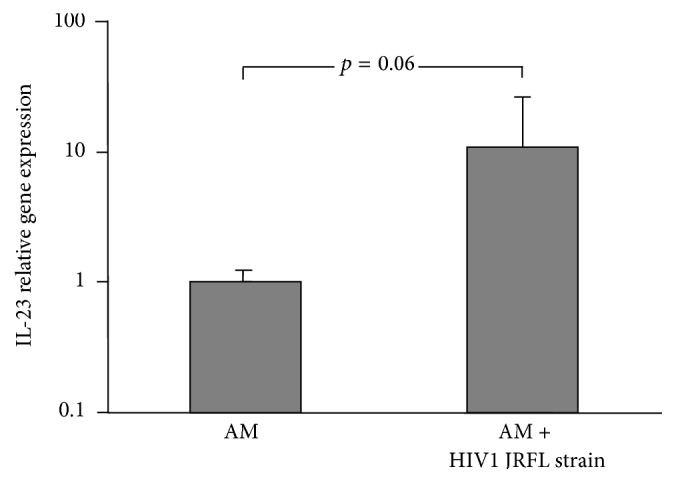
Effect of HIV1 infection* in vitro* on AM-IL-23 upregulation. AM isolated from HIV1^−^ individuals (*n* = 5) were infected with HIV1 JRFL 10^3^ TCID/well or left uninfected. At day 12, RNA was isolated and relative IL-23 expression was assessed by TaqMan PCR (mean ± SEM, *p* = 0.06, Wilcoxon Signed-Rank Test).

To assess the effect of HIV1 infection on AM production of IL-23, we used an established model of* in vitro* infection of AM with an HIV1 laboratory strain, JRFL [30]. IL-23 expression in JFRL-infected AM was compared to IL-23 expression in uninfected control AM. At day 12, IL-23 expression was increased 10-fold compared to uninfected AM, showing a positive trend, but without statistical significance due to high variability associated with JRFL-infected AM (*p* = 0.06, Wilcoxon Signed-Rank Test, Figure 3). This may provide one possible explanation for the increased presence of IL-23 in the ELF of HIV1^+^ individuals independently from their smoking history. This observation is also consistent with the results of experiments in which we measured the levels of IL-23 in conditioned media of AM cultured for 48 hr. Additionally, in a set of experiments with LPS, we also confirmed that IL-23 is inducible in normal AM without HIV1 infection (Supplemental Figure 2). This can explain how, in the setting of chronic inflammation of the airways as in COPD, IL-23 may be present at increased levels, consistent with previously published literature [13, 15, 20, 21]. While our experimental design did not target recruitment of individuals with poorly controlled HIV disease, two available cultures of AM from individuals with measurable viral loads had spontaneous IL-23 release that was 10-fold higher than AM from HIV1^−^ individuals (not shown).

### 3.4. Effect of IL-23 on AM MMP-9 in AM/Lymphocyte Cocultures

**Figure 4:**
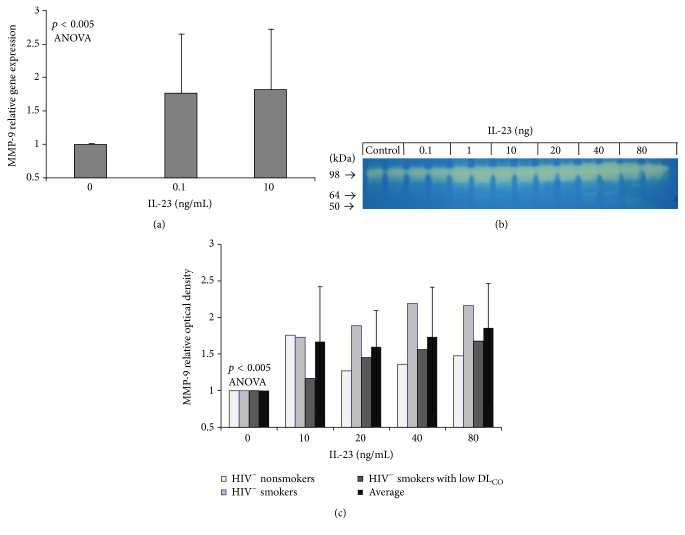
Effect of IL-23 on MMP-9 expression in AM/lymphocyte coculture. (a) Effect of IL-23 (0.1–10 ng/mL) on AM/lymphocyte cocultures (*n* = 13) relative MMP-9 gene expression (mean ± SEM, *p* < 0.005, ANOVA). (b) Gelatin zymography analysis of MMP-9 activity in conditioned media of HIV1^−^ healthy smoker AM/lymphocyte cocultures stimulated with IL-23. Shown is a representative gel. Lanes 1, 2: control; lanes 3, 4: IL-23, 0.1 ng/mL; lanes 5, 6: 1 ng/mL; lanes 7, 8: 10 ng/mL; lanes 9, 10: 20 ng/mL; lanes 11, 12: 40 ng/mL; and lanes 13, 14: 80 ng/mL. (c) Quantitation of gelatin zymography analysis of MMP-9 activity in conditioned media from 10 HIV1^−^ nonsmokers (*n* = 10), HIV1^−^ smokers (*n* = 9), and HIV1^−^ smokers with low DLCO (*n* = 5) after stimulation with IL-23 (0–80 ng/mL; mean ± SEM, *p* < 0.001 ANOVA).

The gelatinase MMP-9 is capable of degrading extracellular matrix proteins and also can activate a number of bioactive molecules such as chemokines that are not a part of the matrix [38, 39]. Based on the data shown in Figure 1 and our prior observations that expression of AM MMP-9 is significantly increased in both HIV1^**+**^ smokers with low DL_CO_ and HIV1^−^ smokers with low DL_CO_ compared to both HIV1^−^ healthy smokers and HIV1^−^ healthy nonsmokers [8], we analyzed the effect of IL-23 on AM MMP-9 production. We used AM/lymphocyte cocultures obtained from HIV1^−^ healthy smokers, stimulated the cultures with IL-23, and then analyzed the relative expression of MMP-9 mRNA with TaqMan RT-PCR. Treatment of AM/lymphocyte cocultures with IL-23 leads to a mild but consistent upregulation of MMP-9 relative gene expression (*n* = 13, *p* < 0.01, ANOVA, Figure 4(a)). To assess whether IL-23 causes increased secretion of MMP-9, we analyzed the concentrations of MMP-9 in conditioned media via gelatin zymography (Figure 4(b)). Stimulation of AM/lymphocyte cocultures with IL-23 leads to an increase in MMP-9 concentration in conditioned media in a dose-dependent fashion (*n* = 24, *p* < 0.001, ANOVA, Figure 4(c)). This increase in MMP-9 concentration showed a similar pattern in all tested phenotypes including HIV1^−^ nonsmokers, HIV1^−^ smokers, and HIV1^−^ smokers with low DL_CO_.

### 3.5. Analysis of IL-23 Receptor Expression on Alveolar Macrophages and Lymphocytes

**Figure 5:**
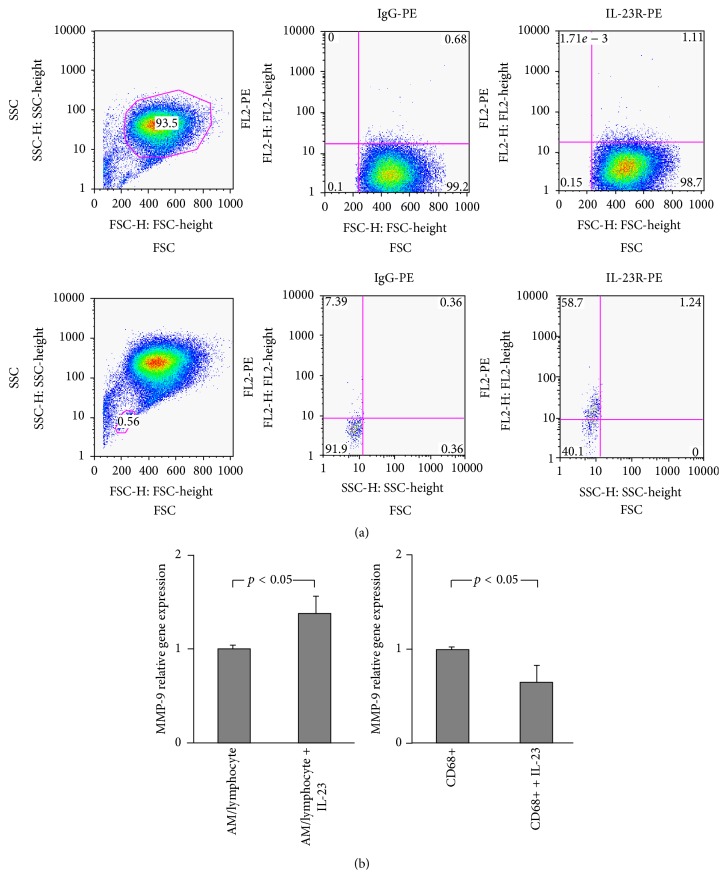
Evaluation of AM and BAL lymphocytes for expression of surface IL-23R. (a) Flow cytometry analysis of cells recovered by lavage from HIV1^−^ smokers with low DLCO. Shown is a representative image from 3 similar experiments. Cells were stained with human IgG-PE (negative control) and mouse anti-human IL-23R-PE antibody 1 : 10. Ordinate: side scatter (SSC). Abscissa: forward scatter (FSC). In the first row, a gated population of AM does not show staining with IL-23R-PE. In the second row, a gated population of lymphocytes shows almost 50% of cells with positive IL-23R staining. (b) CD68 bead-purified AM do not respond to IL-23. Using detachable CD68-labeled magnetic beads, cells recovered by lavage were treated and AM were positively selected based on their surface expression of CD68. Isolated CD68+ cells were cultured and MMP-9 upregulation upon stimulation with IL-23 was compared to cultures of AM purified by adherence only. In contrast to AM purified via the adherence method, CD68+ AM (*n* = 4) do not upregulate MMP-9 upon stimulation with IL-23 (mean ± SEM).

IL-23 is produced by a variety of cell types including AM and its primary target is IL-23 receptor (IL-23R) expressed on lymphocytes. As several reports indicate the presence of IL-23R on alveolar macrophages in diseased lung [40, 41], we investigated the presence and expression of this molecule on normal AM. Using GeneSpring Affymetrix analysis with a *p*-call value of 20% for the threshold for detection of gene expression, we analyzed the AM transcriptome of 87 individuals (24 HIV1^−^ nonsmokers, 34 HIV1^−^ smokers, 18 HIV1^−^ smokers with low DL_CO_, and 11 HIV1^+^ smokers with low DL_CO_). The data demonstrated that the IL-23R was not expressed on AM (not shown). This was consistent with the absence of IL-23R expression in the AM transcriptome of 9 individuals' AM using RNA-Seq (not shown). While IL-23R has been reported to be expressed by AM in sarcoidosis [40], we did not find any evidence of IL-23R expression by AM in normal nonsmokers, HIV1^−^ smokers with early emphysema, or HIV1^+^ individuals. This conclusion was based on an extensive evaluation of IL-23R gene expression (microarray analysis, RNA sequencing, and TaqMan RT-PCR) and flow cytometric analysis of IL-23R expression on the AM. By flow cytometric analysis of freshly isolated cells from BAL, IL-23R was not identified in a gated population of AM, but in a small population of lymphocytes gated by size, IL-23R staining was positive as shown in a single-specimen representative analysis (Figure 5(a)). The absence of IL-23R on the surface of AM implies that the mechanism of IL-23 induced upregulation of MMP-9 is not a consequence of a direct stimulation of IL-23R on the surface of AM, since IL-23R is present neither on AM of HIV1^+^ individuals nor on AM of smokers with low DL_CO_ independent of their HIV1 status.

The response of AM to IL-23 was further evaluated by enrichment of AM in the cultures. Antibodies targeting CD68, a glycoprotein expressed on macrophages but not on lymphocytes, attached to magnetic beads were used to positively select CD68+ AM. After detaching the beads, purified AM were plated on plastic and stimulated with IL-23 in parallel with AM/lymphocyte cocultures obtained by the adherence method. AM purified by immunomagnetic bead separation showed no MMP-9 upregulation upon stimulation with IL-23, in contrast to the effect clearly observed in the AM/lymphocytes obtained by adherence (Figure 5(b)). These data indicate that the downstream effects of IL-23 on MMP-9 upregulation are dependent upon nonmacrophage cells residing in the environment.

### 3.6. The Role of T Cells in IL-23 Induced AM MMP-9 Upregulation

In order to assess the relative proportion of AM in the AM/lymphocyte cocultures obtained by adherence, we analyzed the cultures via flow cytometry and found (1) the proportion of AM was high, similar to or the same as in published literature (>97%); (2) a small, but clearly identifiable population of lymphocytic cells was present in the cultures (not shown). The largest proportion of the nonadherent cells are T lymphocytes as confirmed by positive CD2 and CD4/CD8 staining (data not shown). Memory T cells are known to express IL-23R and to be the primary target for the Th17 modulating effect of IL-23 [40, 42–44]. Using IL-23R antibody for labeling the cells from BAL, we observed that IL-23R is expressed in a population of lymphocytes present in HIV1^−^ and HIV1^+^ smokers with early emphysema. We propose that the effect of IL-23 on cocultures of AM/lymphocytes involves T cells as the primary targets for IL-23.

**Figure 6:**
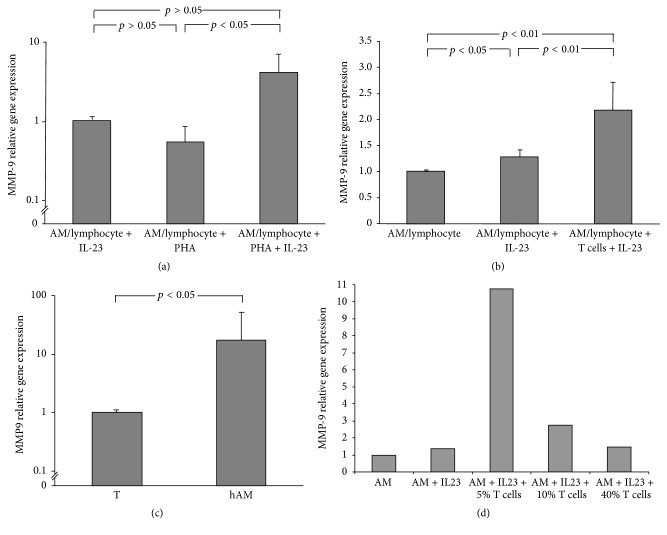
The role of T cells in AM/lymphocyte cocultures in IL-23 induced MMP-9 upregulation. (a) AM/lymphocyte cocultures treated with lymphocyte mitogen PHA (20 *μ*g/mL) respond to IL-23 (10 ng/mL) in a stronger fashion compared to AM/lymphocyte cocultures stimulated with IL-23 only (*n* = 4, mean ± SEM, *p* < 0.05, Kruskall-Wallis). (b) Purified T cells from lavage (isolated via rosetting with neuraminidase treated sheep RBCs) added to AM/lymphocyte cocultures contribute to stronger upregulation of MMP-9 upon IL-23 cytokine stimulation (*n* = 6, mean ± SEM, *p* < 0.01, ANOVA). (c) AM expression of MMP-9 in comparison to its expression in T cells from BAL. T cells were purified from lavage using CD2 magnetic beads and relative expression of MMP-9 was compared by TaqMan PCR (*n* = 11, mean ± SEM, *p* < 0.05, Wilcoxon Signed-Rank Test). (d) The effect of T cells on potentiation of MMP-9 upregulation after IL-23 stimulation (10 ng/mL) of AM/lymphocyte cocultures falls as the number of T cells increases (T cells percentage in AM cultures: 5%, 10%, and 40%; representative results of the effects seen in 4 experiments).

To further assess whether cells other than AM contribute to MMP-9 upregulation, we evaluated the effects of phytohemagglutinin (PHA), a lectin that acts as a specific mitogen for T lymphocytes, on AM/lymphocyte cocultures MMP-9 expression. AM/lymphocyte cocultures treated with PHA alone do not upregulate MMP-9. AM/lymphocyte cocultures stimulated with both IL-23 and PHA had increased MMP-9 expression in comparison to AM/lymphocyte cocultures stimulated with either PHA or IL-23 alone (*p* < 0.05, Kruskall-Wallis, Figure 6(a)).

Based on these data and the fact that T cell-related chemokine levels were elevated in the ELF of HIV1^+^ smokers with low DL_CO_ (Figure 1), we investigated the effect of adding additional T cells on the release of MMP-9 by AM/lymphocyte cocultures stimulated with IL-23. T cells were isolated from BAL fluid via rosetting with neuraminidase-treated sheep RBCs [32]. AM/lymphocyte cocultures obtained by adherence were treated with IL-23 in the presence or absence of purified T cells from the BAL and the resulting MMP-9 expression was analyzed via Taqman RT-PCR. Addition of purified T cells from BAL to AM/lymphocyte cocultures leads to a significantly increased expression of MMP-9 (*n* = 6, *p* < 0.01, ANOVA, Figure 6(b)).

**Figure 7:**
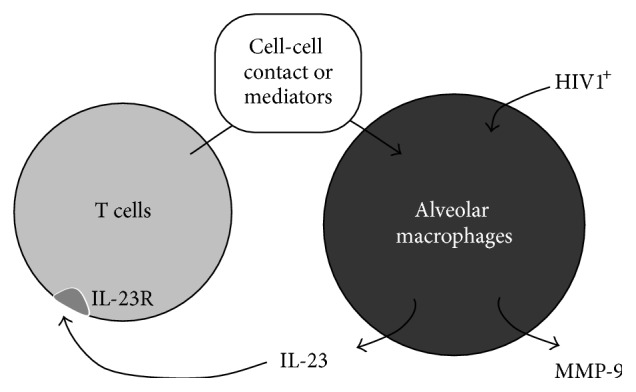
Proposed mechanism of IL-23 induced upregulation of AM MMP-9. IL-23 induced production of MMP-9 is indirect and requires the presence of non-AM cells such as T cells (“T”) in the alveolar environment. Memory T cells may express IL-23R and are known to be the target of IL-23. Upon stimulation with IL-23, T cells further induce the upregulation of MMP-9 in AM by a mechanism that likely requires direct cell-to-cell contact between T cells and AM.

Since MMP-9 is produced by blood T cells [45], we compared the gene expression of MMP-9 in two cell types used in this experiment, AM and BAL T cells purified via CD2-labeled magnetic beads. While BAL T cells did produce some MMP-9, there was greater than 1 log higher MMP-9 expression in AM in comparison to alveolar T cells (*p* < 0.05, Wilcoxon Signed-Rank Test, Figure 6(c)). In addition, the T cell effect on MMP-9 upregulation is unlikely to be based on T cell MMP-9 expression, because as the percentage of T cells in AM cultures was increased from 5% to 10% and to 40% of total cells MMP-9 expression decreased (Figure 6(d)). Thus, higher percentages of T cells in cultures of AM at the time of harvesting RNA may have simply “diluted” the overall MMP-9 expression in these cultures as T cells express lower levels of MMP-9 mRNA in comparison to AM as discussed above. Alternatively, T cells may exert a biphasic effect on AM MMP-9 expression. While we cannot exclude a possible contributing role from other cell types such as NK cells or neutrophils, these results show that T cells, while not a major direct contributor to the expression of MMP-9, may represent an important target for IL-23 and may significantly affect overall upregulation of MMP-9 in AM-T-lymphocyte cocultures. Taken together, these observations suggest that, upon stimulation with IL-23, T cells interact with AM leading to the upregulation of AM MMP-9 via a T lymphocyte-AM interaction (Figure 7).

## 4. Discussion

Increased AM and ELF MMP-1 and MMP-9 expression have been linked to emphysema in humans and in animal models [7, 22, 38, 46, 47]. Yearsley et al. [48] were the first to observe increased lung tissue expression of MMP-9 in autopsies of HIV1^+^ individuals with emphysema who died of AIDS. HIV1^+^ smokers with emphysema have both upregulated AM expression of MMPs and upregulated and activated MMPs in ELF compared to HIV1^−^ smokers with emphysema [8]. HIV1^+^ individuals are also known to have increased lung cytokine expression [5, 7, 9–12]. Since several cytokines can upregulate monocyte-derived macrophage MMP expression, it is not unreasonable to hypothesize that these observations may all be directly related [22, 45–47]. Our cytokine array data indicating substantial upregulation of lung cytokines in HIV1^**+**^ individuals are consistent with the broad upregulation of ELF cytokines observed by others [9–12]. Interestingly, in the ELF cytokine profiles, HIV1^−^ smokers with low DL_CO_ displayed detectable Th17 related cytokines as a common feature with HIV1^+^ nonsmokers and HIV1^+^ smokers with low DL_CO_. This increase is not a feature of the cytokine profile of HIV1^−^ healthy nonsmokers or HIV1^−^ healthy smokers. The presence of a group of proinflammatory cytokines in the ELF of HIV1^+^ nonsmokers which are similar to the cytokine profile in the ELF of HIV1^−^ smokers with low DL_CO_ likely plays an important role in the premature development of emphysema in HIV1^+^ individuals who smoke.

The observation of increased Th17 cytokines in the ELF of HIV1^+^ smokers with low DL_CO_ is not surprising, as there is emerging evidence of the relevance of Th17 cellular responses in lung pathology [13, 49–51]. The Th17 cellular immune response has been described with recent modification of the Th1/Th2 paradigm based on the identification of IL-23 and IL-17 involvement in autoimmune diseases [44, 52–54]. Th17 cells, characterized by the release of effector-cytokines IL-17, IL-22 and the presence of regulatory-cytokines such as IL-23, are functionally distinct from Th-1 cells and Th-1-related cytokines such as IL-12 and IFN-*γ* [52]. Depending on the timing, the tissue, and the local microenvironment, IL-17-secreting cells appear to play both beneficial and detrimental roles in lung immunity and disease [55]. Th17 cells may have a protective role in host defenseagainst certain extracellular bacteria and fungi [44, 51, 55, 56]. Th17 cells are localized at the mucosal level, including the gut and lung mucosa, and their cytokine production adds to the first line of defense against infectious agents [57].

Th17 cells and related effector cytokines may also play a prominent role in the pathogenesis of inflammatory lung diseases, where they are hypothesized to promote tissue destruction [50, 51]. Given the significant increase in Th17 related cytokines in stable COPD, it has been inferred that Th17 cellular responses may be driving the chronic inflammation seen in COPD [13, 58]. Mice exposed to cigarette smoke exhibit enhanced IL-17 production [59], while IL-17 receptor knockout mice are protected from emphysema induced by cigarette smoke exposure [13]. Patients with stable COPD exhibited elevated numbers of IL-22 and IL-23 positively staining cells in the bronchial epithelium and IL-17 positive cells in the submucosa [15]. In ex-smokers with mild to moderate COPD, the number of IL-17^+^ cells in the bronchial submucosa is increased compared with nonsmoking control subjects [60]. In the same study, a significant, albeit small, increase in sputum IL-17 has been correlated with airflow obstruction and lung function impairment, but not airway inflammation [60].

Th17 cells are also relevant in HIV1 infection as deterioration of the Th17 lineage may correlate to the rapid progression of the disease in infected individuals [17]. In HIV1 infection, Th17 cells are depleted from the gut but remain present in blood and lung [61]. Increased susceptibility of HIV1-infected individuals to opportunistic pathogens targeting the respiratory tract such as* Pneumocystis carinii* and* M. tuberculosis* for which Th17 cells exhibit antigenic specificity suggests that a dysfunction of Th17 cellular responses may correlate with progression of the disease [17, 62, 63].

IL-23 is one of the key cytokines characterizing the Th17 immune response and it represents an important link between the innate and adaptive immune responses [44, 64]. It is a heterodimeric molecule consisting of the p40 subunit shared in common with IL-12, combined with a unique p19 subunit [65]. While both cytokines can induce IFN-*γ* expression in CD4+ cells, IL-23, although not crucial for Th17 differentiation, plays an important role in maintaining Th17 effector function [66]. IL-23 is expressed mostly by activated macrophages and dendritic cells while its receptor, IL-23R (the receptor for the p19 subunit), is expressed in memory T cells of the Th17 subtype [43]. IL-23 deregulation may lead to inability to adequately protect the organism from certain pathogens while its overexpression may be linked to various autoimmune phenomena [58, 67]. IL-23 gene expression is increased in patients with COPD as compared to nonsmokers and, in an animal model, cigarette smoking increases the lung expression of IL-23 [68]. Increased levels of IL-23 correlate with increased levels of MMPs as detected in mouse models such as TLR4 knock-out mice [69] and in human peripheral blood of patients with relapsing-remitting multiple sclerosis [70]. In support of this concept, intranasal administration of IL-23 protein to mice resulted in increased lung MMP-9, a protease linked to the influx of inflammatory cells into the lung in COPD as well as the destruction of lung tissue in emphysema [21, 63]. Based on this background, we hypothesized that, in the lower respiratory tract in HIV1^+^ smokers, increased IL-23 is acting on T-lymphocytes to induce increased AM MMP-9 gene expression and consequently ELF MMP-9 protein levels.

There are multiple pathways by which chronic inflammation may lead to upregulation of MMP-9. Proinflammatory cytokines such as IL-1*β*, TNF-*α*, and IL-6 are well known to be inducers of MMP-9 expression in monocyte-derived macrophages [47, 70, 71]. Data addressing the effect of IL-23 on production of MMPs are very limited. IL-23 has previously been shown to upregulate MMP-2* in vivo* in the ileum of mice infected with* Toxoplasma gondii* [72]. More specifically for the lung, intranasal application of IL-23 in a mouse model increased MMP-9 levels in ELF and induced IL-17 mRNA expression [21]. Our data support the hypothesis that increased ELF levels of IL-23 may be responsible, at least in part, for the increased ELF MMP-9* in vivo.* A limitation of our study is the fact that the observed increase in MMP-9 in the lungs of HIV1^+^ smokers with low DL_CO_ may be a consequence of increased IL-23 in the ELF, but causality has been proven only* in vitro*.

Memory T cells are known to express IL-23R and to be the primary target for the Th17 modulating effect of IL-23 [40, 42, 44]. We propose that the effect of IL-23 on cocultures of AM/lymphocytes involves T cells as the primary targets for IL-23.

Our observations suggest that, upon stimulation with IL-23, T cells interact with AM leading to the upregulation of AM MMP-9. There are several possible mechanisms that might explain this relationship between AM and T cells. Given its identification in ELF in the same phenotypes in which IL-23 is present, IFN-*γ* was evaluated as a potential target cytokine. While IFN-*γ* is indeed upregulated in T cells upon IL-23 stimulation consistent with previously reported data [73] and confirmed by our* in vitro* experiments, IFN-*γ* does not induce upregulation of MMP-9 in human AM (not shown). In addition, we were unable to passively transfer the effect on AM MMP-9 upregulation by adding conditioned media from T cell cultures stimulated with IL-23. This observation suggests that that direct cell/cell contact may be required. The relevance of the T-lymphocyte/monocyte interaction for the induction of MMP-9 expression has been described [6, 74, 75]. Various stimuli induce T-lymphocytes to activate monocytes indirectly via released cytokines and directly via direct cellular contact that may include antigen recognition on antigen specific T cell clones, cross-linking of surface receptors, or membrane-associated cytokines [76]. In addition to membrane-associated cytokines, other surface molecules have been assessed for their ability to activate monocytes upon contact with stimulated T-cells, for example, leukocyte function antigen- (LFA-) 1/intercellular adhesion molecule-1, CD2/LFA3, CD40/CD40L, and lymphocyte activation-antigen-3 [76]. CD40 is a costimulatory protein found on antigen presenting cells and is required for their activation. The binding of CD154 (CD40L) on Th cells to CD40 activates antigen presenting cells and induces a variety of downstream effects. CD40-ligand (CD-40L) is a member of the tumor necrosis factor (TNF) superfamily and activated CD4+ T cells are the predominant CD40L expressing population [77]. IL-23 administration to peripheral blood T cells* in vitro* upregulated CD40L expression (not shown). One possibility is that this upregulation of CD40L may lead to enhancement of the direct T cell-AM activation which may further induce the upregulation of MMP-9 in AM.

## 5. Conclusions

Our data support the hypothesis that IL-23 upregulates MMP-9 in the lung. This effect is indirect and requires both AM and T lymphocytes. Taken together, these data suggest that the Th17 pathway may play a role in the early development of emphysema in HIV1^+^ individuals.

## Supplementary Material

Supplemental Figure 1. ELF cytokine profiles. See Supplemental Table I for list of cytokine abbreviations. Concentrated ELF was analyzed with the Proteome Profiler™ Human Cytokine Array Panel A A. HIV1- healthy nonsmokers (n=6), B. HIV1- healthy smokers (n=7), C. HIV1- smokers with low DLCO (n=6); D. HIV1+ nonsmokers (n=4), and E. HIV1+ smokers with low DLCO (n=4).
Supplemental Figure 2. IL-23 is inducible in normal AM without HIV1 infection. The graph represents the average upregulation of IL-23 expression in four different experiments (t-Test, p=0.09).
Supplemental Table I. Cytokine Nomenclature.

## Figures and Tables

**Table 1:** Study population and alveolar macrophage samples^(1)^.

Parameter	Healthy HIV1^−^ nonsmoker	HIV1^+^ nonsmoker^(7)^	Healthy HIV1^−^ smoker	HIV1^−^ smoker with low DLCO	HIV1^+^ smoker with low DLCO^(8)^
*N*	6	4	7	6	4
Gender (male/female)	5/1	2/2	3/4	6/0	2/2
Age (yr)	41 ± 2	44 ± 6	44 ± 2	47 ± 2	46 ± 1
Race (B/W/O)^(2)^	4/1/1	2/1/1	3/2/2	6/0/0	4/0/0
Smoking history (pack-yr)	0	0	31 ± 8	48 ± 9	27 ± 7
Urine nicotine (ng/mL)^(3)^	Negative	Negative	1328 ± 623	1817 ± 566	813 ± 307
Urine cotinine (ng/mL)^(3)^	Negative	Negative	973 ± 273	1277 ± 438	1419 ± 561
Blood carboxyhemoglobin (%)^(4)^	0.5 ± 0.4	0	2.6 ± 1.5	1.2 ± 0.3	2.0 ± 0.8
Pulmonary function parameters^(5)^					
FVC	103 ± 3	104 ± 11	110 ± 5	108 ± 5	103 ± 7
FEV1	107 ± 2	105 ± 11	106 ± 6	108 ± 2	115 ± 8
FEV1/FVC	85 ± 1	83 ± 2	80 ± 2	80 ± 2	83 ± 2
TLC	91 ± 4	102 ± 9	95 ± 3	88 ± 3	90 ± 6
DLCO	92 ± 5	91 ± 3	99 ± 5	68 ± 4	69 ± 5
BAL cells^(6)^					
Total number recovered (×10^7^)	2.1 ± 0.4	3.3 ± 0.8	2.9 ± 1.0	3.3 ± 0.8	3.7 ± 0.9
% viability	92.0 ± 2.0	80.4 ± 8.6	95.3 ± 1.0	93.1 ± 1.5	87.3 ± 3.5
% lymphocytes	5.5 ± 1.3	6.9 ± 2.4	2.6 ± 0.6	2.3 ± 0.5	13.0 ± 5.2
% alveolar macrophages	91.7 ± 1.7	89.8 ± 2.9	94.6 ± 1.2	97.2 ± 0.8	87.5 ± 4.0
% polymorphonuclear cells	1.0 ± 0.3	1.7 ± 0.7	1.2 ± 0.6	0.5 ± 0.2	0.5 ± 0.2
% epithelial cells	0.8 ± 0.5	1.7 ± 0.3	0.3 ± 0.32	0.3 ± 0.1	0.7 ± 0.4

^(1)^Demographic characteristics of subjects; bronchoalveolar lavage cell differentials represent the samples used to obtain the data; all data are mean ± standard error.

^(2)^B = black; W = white; O = other.

^(3)^Urine nicotine and cotinine > 200 = active smoker, 50–200 = passive smoker; <50 nonsmoker; data represent the mean of two determinations on the days of screening and bronchoscopy.

^(4)^Blood carboxyhemoglobin is a secondary marker of current smoking status; nonsmokers < 1.5%.

^(5)^Pulmonary function parameters included: FVC, forced vital capacity (% predicted); FEV1, forced expiratory volume in 1 sec; FEV1/FVC (% observed); TLC, total capacity (% predicted); DLCO, diffusion capacity (% predicted).

^(6)^BAL = bronchoalveolar lavage; shown is the cell differential prior to alveolar macrophage purification.

^(7)^Individual plasma HIV1 viral loads: <50 (*n* = 2); 16,000; unknown (*n* = 1). Individual CD4 count: 700; 696; 450; unknown (*n* = 1).

^(8)^Individual plasma HIV1 viral loads: <50 (*n* = 2); 8000; unknown (*n* = 1). Individual CD4 count: 384; 1070; 494; unknown (*n* = 1).
