# Supplementary material for: The Role of Interleukin-23 in the Early Development of Emphysema in HIV1+ Smokers
Source: J Immunol Res. 2016 Jun 29;2016:3463104. doi: 10.1155/2016/3463104 (PMC4942665; doi:10.1155/2016/3463104)
Supplement: Supplementary file 1 — Supplemental Figure 1. ELF cytokine profiles. See Supplemental Table I for list of cytokine abbreviations. Concentrated ELF was analyzed with the Proteome Profiler™ Human Cytokine Array Panel A A. HIV1- healthy nonsmokers (n=6), B. HIV1- healthy smokers (n=7), C. HIV1- smokers with low DLCO (n=6); D. HIV1+ nonsmokers (n=4), and E. HIV1+ smokers with low DLCO (n=4). Supplemental Figure 2. IL-23 is inducible in normal AM without HIV1 infection. The graph represents the average upregulation of IL-23 expression in four different experiments (t-Test, p=0.09). Supplemental Table I. Cytokine Nomenclature. [file 3463104.f1.pdf]

Supplemental Table I. Cytokine Nomenclature[1]

Symbol	Alternate name	Name
G-CSF	CSFβ	Granulocyte colony-stimulating factor
GM-CSF	CSFα, CSF-2	Granulocyte-macrophage colony stimulating factor
IFNγ	Type II IFN	Type II interferon
IL-2		Interleukin-2
IL-4		Interleukin-4
IL-5		Interleukin-5
IL-6		Interleukin-6
IL-10		Interleukin-10
IL-13		Interleukin-13
IL-1F1	IL-1α	Interleukin-1 alpha
IL-IF2	IL-1β	Interleukin-1 beta
IL-1F3	IL-1ra	Interleukin-1 receptor antagonist
TNFα		Tumor necrosis factor-alpha
IP-10	CXCL10	Chemokine (C-X-C motif) ligand 10
IL-8	CXCL8	Interleukin-8
GROα	CXCL1	Growth-related oncogene alpha
MCP-1	CCL2	Chemokine (C-C motif) ligand 2
CCL3	MIP-1α	Chemokine (C-C motif) ligand 3
CCL4	MIP-1β	Chemokine (C-C motif) ligand 4
RANTES	CCL5	Chemokine (C-C motif) ligand 5
I-TAC	CXCL11	Chemokine (C-X-C motif) ligand 11
SDF-1	CXCL12	Chemokine (C-X-C motif) ligand 12
I-309	CCL1	Chemokine (C-C motif) ligand 1
IL-12p70		Interleukin-12p70
IL-32α		Interleukin-32 alpha
IL-16	LCF	Interleukin-16
IL-23		Interleukin-23
IL-27		Interleukin-27
IL-17	IL-17A	Interleukin-17
IL-17E	IL-25	Interleukin-17E
slCAM-1	CD54	Soluble intercellular adhesive molecule-1
MIF	GIF	Macrophage inhibition factor
PAI-1	SERPINE1	Plasminogen activator inhibitor-1
C5a	C5	Complement Component 5a
CD40 L	CD154	CD40 ligand
sTREM-1		Soluble triggering receptor expressed on myeloid cells 1

[1] Listed are the cytokines in the Proteome Profiler, Human Cytokine Array Panel A (R&D Systems, Minneapolis, MN).

Supplemental Figure Legends

Supplemental Figure 1. ELF cytokine profiles. See Supplemental Table I for list of cytokine abbreviations. Concentrated ELF was analyzed with the Proteome Profiler™ Human Cytokine Array Panel A **A.** $HIV1^-$ healthy nonsmokers (n=6), **B**. $HIV1^-$ healthy smokers (n=7), **C.** $HIV1^-$ smokers with low DLCO (n=6); **D.** $HIV1^+$ nonsmokers (n=4), and **E.** $HIV1^+$ smokers with low DLCO (n=4).

Supplemental Figure 2. IL-23 is inducible in normal AM without HIV1 infection. The graph represents the average upregulation of IL-23 expression in four different experiments (t-Test, p=0.09)

Supplemental Figure 1

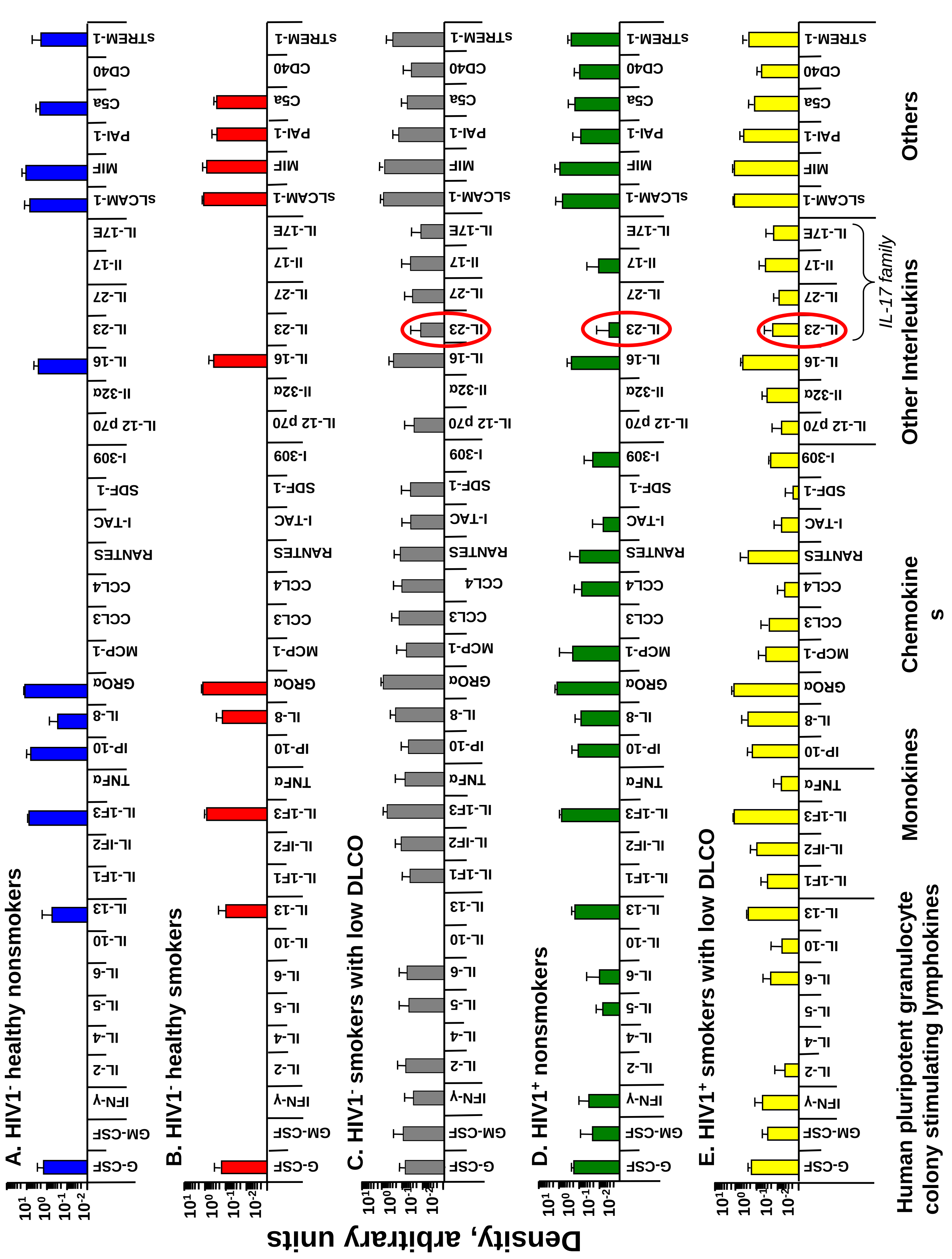

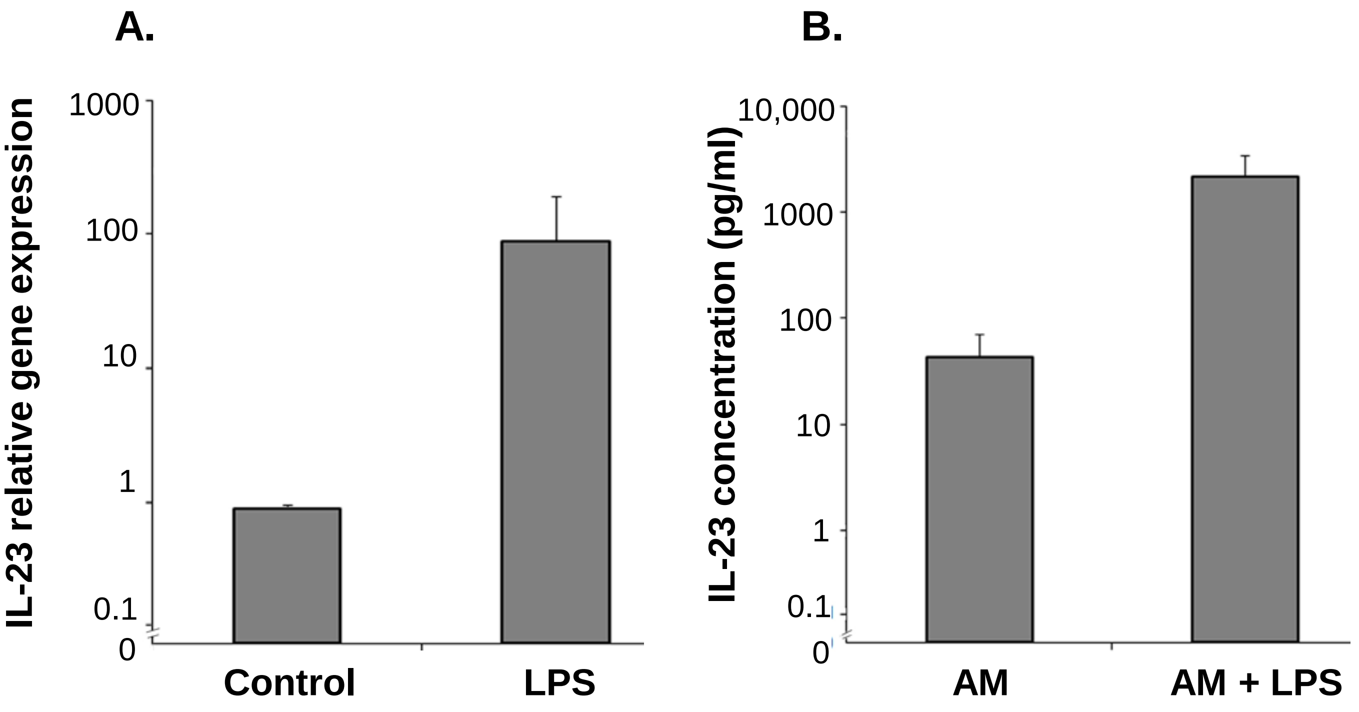
A.
B.
1000
100
10
1
0.1
0
IL-23 relative gene expression
Control
LPS
10,000
1000
100
10
1
0.1
0
IL-23 concentration (pg/ml)
AM
AM + LPS
